# A Comparative Study of Protocols for Mouse Embryonic Stem Cell Culturing

**DOI:** 10.1371/journal.pone.0081156

**Published:** 2013-12-10

**Authors:** Christoffer Tamm, Sara Pijuan Galitó, Cecilia Annerén

**Affiliations:** 1 Department of Medical Biochemistry and Microbiology, Uppsala University, Uppsala, Sweden; 2 GE Healthcare Bio-Sciences AB, Uppsala, Sweden; Brigham and Women's Hospital, United States of America

## Abstract

Most stem cell laboratories still rely on old culture methods to support the expansion and maintenance of mouse embryonic stem (ES) cells. These involve growing cells on mouse embryonic fibroblast feeder cells or on gelatin in media supplemented with fetal bovine serum and leukemia inhibitory factor (LIF). However, these techniques have several drawbacks including the need for feeder-cells and/or use of undefined media containing animal derived components. Culture of stem cells under undefined conditions can induce spontaneous differentiation and reduce reproducibility of experiments. In recent years several new ES cell culture protocols, using more well-defined conditions, have been published and we have compared the standard culture protocols with two of the newly described ones: 1) growing cells in semi-adherence in a medium containing two small molecule inhibitors (CHIR99021, PD0325901) and; 2) growing cells in a spheroid suspension culture in a defined medium containing LIF and bFGF. Two feeder-dependent mouse ES (mES) cell lines and two cell lines adapted to feeder-independent growth were used in the study. The overall aim has not only been to compare self-renewal and differentiation capacity, but also ease-of-use and cost efficiency. We show that mES cells when grown adherently proliferate much faster than when grown in suspension as free-floating spheres, independent of media used. Although all the tested culture protocols could maintain sustained pluripotency after prolonged culturing, our data confirm previous reports showing that the media containing two chemical inhibitors generate more pure stem cell cultures with negligible signs of spontaneous differentiation as compared to standard mES media. Furthermore, we show that this medium effectively rescues and cleans up cultures that have started to deteriorate, as well as allow for effective adaption of feeder-dependent mES cell lines to be maintained in feeder-free conditions.

## Introduction

A key focus for scientists in the embryonic stem (ES) cell research field is maintaining cells in an undifferentiated and proliferative state without causing chromosomal aberrations or loss of pluripotency. When the first mouse ES (mES) cell lines were established [1], [2] in 1981, the cells were grown on pre-plated mitotically inactivated mouse embryonic fibroblast (MEF) feeder cells in media supplemented with selected batches of fetal bovine serum (FBS) and/or conditioned media from teratocarcinoma stem cell cultures. The feeder cells provide a matrix that support mES cell attachment and secrete various growth factors that enhance the survival and propagation of mES cell growth [3], [4] whereas FBS provides hormones and essential nutrients, as well as altering the physiological/physiochemical properties of the medium. It was later discovered that a single cytokine, leukemia inhibitory factor (LIF), could retain self-renewal and pluripotency of mES cells in the absence of feeder cells [5], [6]. Culture of mES cells on MEFs in FBS- and LIF-containing media is still the standard protocol used in many laboratories although some mES cell lines have been adapted to grow in feeder-free cultures on gelatinized surfaces with media supplemented with serum and LIF [7], [8]. These cell culture protocols have the shortcoming that many of their components (e.g. FBS, BSA, gelatin) are not fully defined and are animal-derived. FBS, for instance, contains various growth factors and other undefined components that promote mES cell growth, but it has also been suggested to contain potential differentiation factors [9] that can affect mES cell plating efficiency, growth and differentiation. Therefore FBS batches need to be pre-screened and ES-qualified to ensure that the net-effect of serum factors that sustain mES cell maintenance and growth outweighs the effects of differentiation-inducing factors. In addition, feeders secrete a plethora of factors impossible to control and are a possible source of pathogenic contamination. To improve control over what factors mES cells are actually subjected to and to avoid interference from undesired factors, several newer and more well-defined protocols have been established. In 2003 it was shown that BMP4 could efficiently be used in combination with LIF for mES cell derivation and maintenance in serum- and feeder-free cultures by suppressing neural differentiation via the induction of Id proteins through the Smad pathway [10]. In 2004, a chemically defined (the exact formulation is not described) synthetic knockout serum replacement (KOSR) was developed to replace serum [11]. However, KOSR cannot alone support mES single-cell culture in the absence of feeders, and a recent study shows that, similar to FBS, it exhibits considerable lot-to-lot variability [12]. In 2008, it was shown that mES cells could be maintained in the absence of serum and feeder cells as free-floating spheres in a N2 supplemented medium with LIF and bFGF (herein named ESN2) [13], [14]. In contrast to previously reported ES cell sphere cultures in media supplemented with B27 [15], the spheres grown in ESN2 do not express the neural stem cell marker nestin. However, this protocol has been reported to render mES cells prone to neurogenic differentiation [13], [14]. Recently, a defined media supplemented with two inhibitors, the mitogen-activated protein kinase (MAPK)/extracellular-signal-regulated kinase (ERK) kinase (MEK) inhibitor PD0325901 and the glycogen synthase kinase 3 (GSK3) inhibitor CHIR99021, added to a B27 and N2 supplemented medium (herein named 2i) was shown to maintain mES cell self-renewal without the addition of exogenous factors [16]. PD0325901 inhibits the autocrine stimulation of the mitogen-activated protein kinase (ERK1/2) pathway by fibroblast growth factor-4 (FGF4), which has been shown to be elemental for ES cell differentiation [17], [18]. GSK3 inhibition impairs the endogenous repressor activity of Tcf3, a transcriptional repressor of the core pluripotency network [19]. Mouse ES cells cultured in 2i medium still respond to LIF, which enhances cloning efficiency and proliferation rates. In fact, the use of 2i medium plus LIF has enabled the isolation of ES cells from previous in-compatible mouse strains as well as for the first time from rats [20], [21], [22], [23].

In the present study we have compared mES cells cultured on feeders, gelatin and in suspension in standard in-lab made medium containing ES-qualified serum and KOSR (SCM), an equivalent commercially available complete medium (mES Prime Kit), and the serum-free ESN2 and 2i media. The aim has not only been to compare proliferation rates, self-renewal and differentiation capacity, but also to evaluate ease-of-use, and time and cost efficiency. This report demonstrates properties and trade-offs of different mES cell culture techniques as well as provides assistance to novices in the stem field in their choice of culturing techniques. Our results further confirm that serum-free and feeder-free culture of mES cells holds several advantages over the classical serum-containing feeder-dependent culture methods, including improved effectiveness of maintaining ES cells in an undifferentiated stage. Moreover, our results show that the use of 2i media can rescue and “clean up” mES cultures that had started to differentiate, corroborating similar findings using a predecessor of 2i (3i) [23]. Finally we have identified a novel use of the 2i media for adaption of feeder-dependent mES cell lines to be maintained in feeder-free conditions.

## Materials and Methods

### Embryonic stem cell culturing

Four different culture media were used in this study: 1) SCM [24], consisting of Glasgow modification of Eagles medium (GMEM) containing 5% ES-qualified fetal calf serum, 5% KnockOut^TM^ serum replacement and 1,000 U/ml LIF (Millipore); 2) mES Prime Kit, a commercially available LIF- and FBS-supplemented complete media (PAA Laboratories/GE Healthcare); 3) 2i medium [25], a serum-free N2B27 medium supplemented with MEK inhibitor PD0325901 (1 μM) and GSK3 inhibitor CHIR99021 (3 μM) (both from Selleckchem), and 1,000 U/ml LIF (Millipore) and; 4) ESN2 [13], consisting of DMEM/F12 supplemented with N2, 10 ng/ml bFGF (R&D Systems), and 1,000 U/ml of LIF. Feeder dependent R1 [26] and C57 [27] mES cells were cultured on irradiated MEFs (R&D Systems). Feeder depletion was achieved by plating dissociated cells for 5–15 min at 37°C. ES cells were recovered from the supernatant and reseeded to a new plate. This procedure was repeated for three consecutive times. The feeder-independent E14 mES cell line and E14/T, a polyoma large T-constitutively expressing mES cell line, were cultured on gelatin as previously described [7]. For adherent growth cells were cultured in SCM, mES Prime Kit or 2i medium with or without 2% FBS. For suspension growth, all four cell lines were cultured as spheres in either 2i or ESN2 media. Trypsin EDTA was used to passage adherent cells and TrypLE^TM^ Express (Life Technologies) was used to passage suspension cultures. For all culture conditions described above the cells were cultured for at least 15 passages, under which proliferation rates were assessed over 5 consecutive passages between passages 6–10. At passage 10 the cells were frozen down and thawed, and at passage 15 the cells where karyotyped, analysed for AP-activity, harvested for qPCR analysis or induced to differentiate to assess for pluripotency. Suspension-grown aggregates were cryosectioned at passage 10 and stained for Ki67 and Oct4 to assess proliferation and self-renewal throughout the spheres. To induce spontaneous differentiation, cells were maintained on gelatin in SCM in the absence of LIF for 6 days. Differentiation was verified by qPCR analysis to confirm a distinct down-regulation of mES cell specific genes, and pluripotency was assessed by the up-regulation of genes specifically expressed in each of the three germ layers.

### Quantitative real-time polymerase chain reaction (qPCR)

Total RNA was extracted and purified with the GenElute^TM^ mammalian total RNA miniprep kit (Sigma-Aldrich) according to the manufacturer's instruction. First-strand cDNA was produced according to the manufacturer's protocol with iScript^TM^ cDNA synthesis kit (Bio-Rad) using 1 μg RNA. Quantitative real-time PCR was performed according to the manufacturer's instructions using the SsoFast^TM^ EvaGreen^TM^ supermix on the Miniopticon™ Real-Time PCR Detection System (both from Bio-Rad). The average C(t) value for each gene was normalized against 18S and the comparative C(t) value (fold change) was calculated using the 2− ΔΔC(t) formula. To assess whether there was any significant difference between the various culturing media, the comparative Ct value means were calibrated for each cell line and then pooled together in order to filter out in-sample variations. Primers used were:

18S (forward: AGTCCCTGCCCTTTGTACACA, reverse: GATCCGAGGGCCTCACTAAAC), Oct4 (forward: GATGCTGTGAGCCCAAG-GCAAG, reverse: GGCTCCTGATCAACAGCATCAC), FGF4 (forward: GAGGCGTGGTGAGCATCTT, reverse: ACACTCGGTTCCCCTTCTTG), Brc (forward: TGTGGCTGCGCTTCAAGGAGC, reverse: GTAGACGCAGCTGGGCGCCTG), Actc1 (forward: CCAAAGCTGTGCCAGGATGT, reverse: GCCATTGTCACACACCAAAGC), Dab2 (forward: TGAAGCAGACAGCCAGAACA, reverse: CAACAGACAAGGATTTGATAGGG), Gata6 (forward: GAAGCGCGTGCCTTCATC, reverse: GTAGTGGTTGTGGTGTGACAGTTG), FGF5 (forward: AAAGTCAATGGCTCCCACGAA, reverse: CTTCAGTCTGTACTTCACTGG), Pax6 (forward: TAACGGAGAAGACTCGGATGAAGC, reverse: CGGGCAAACACATCTGGATAATGG), Sox17 (forward: CCCAACACTCCTCCCAAAGTATC, reverse: TTCCCTGTCTTGGTTGATTTCTC), Nestin (forward: CTCTTCCCCCTTGCCTAATACC, reverse: TTTAGGATAGGGAGCCTCAGACAT).

### Proliferation assay

1×10^5^ mES cells/well, were seeded in 24-well plates and cultured for 48 hrs. The cells were then trypsinized into single cell suspension and automatically counted using the TC10™ Automated Cell Counter (Bio-Rad). To avoid quantification and seeding of MEFs the mES cells grown on MEFs were feeder-depleted at each passage. Proliferation rates, i.e. cell doubling times (DT), was calculated using the following formula: DT =  (t−t_0_) log2/(logN−logN_0_) where t, t_0_ indicate time points at counting and initial plating, respectively; and N, N_0_ indicate number of cells at respective time points. Results are presented as mean doubling times ± SD of 5 consecutive passages.

### Alkaline-phosphatase (AP) colouring assay and nuclear morphology

Cells were fixed with 4% paraformaldehyde (PFA) for 1 min at room temperature (RT), stained with Vector Red alkaline phosphatase substrate kit (Vector laboratories) according to manufacturer's instruction, and examined under phase contrast microscope.

### Transfections and luciferase reporter assay

Reporter constructs and the pCMV β-gal reference plasmid containing a bacterial β-galactosidase gene were introduced into the mouse ES cells by transfection with Lipofectamine™ 2000 (Life Technologies) according to the manufacturer's recommendations (final concentration 1 μg DNA/well). In the present study we used pGL3-basic vectors carrying the 2.1 kb upstream region of the mouse Oct-3/4 gene (the upstream end at nt. −2136 relative to the translational start site) [28] or the 1 kb upstream region of mouse Nanog (the upstream end at nt. −983 relative to transcription start site) [29], pCS GT-IIC-luciferase (GTIIC) [30], pCMV β-gal and pmaxGFP. Cells were incubated at 37°C for 4 hours, after which fresh cell medium was added. 24 hours after transfection cells were harvested and lysed, and extracts were assayed for luciferase and β-galactosidase activities in a microplate luminometer and photometer reader (Wallac VICTOR 1420 Multilabel Counter: Perkin Elmer). Results are presented as the mean percentage of the control (n = 3).

### Chromosomal counting

Cells were exposed to 100 μM Demecolcine for 2 hrs prior to trypsination and harvest. Cells were then incubated in 37°C 0.56% KCl swelling solution for 5 minutes, and subsequently fixed using methanol-acetic acid fixative (3∶1) for 15 min at 4°C. The cell suspension was dropped onto semi-dry cold glass slides from an altitude of around 50 cm to ensure cell breakage. After 1 hr drying at room temperature, cells were stained with Giemsa in dH_2_O (1∶20) for 10 minutes before chromosomal counting using phase contrast microscopy.

### Immunocytochemistry

Cryosections of supension spheroids were fixed with cold 4% PFA (Sigma-Aldrich) for 60 minutes and then washed with PBS. Primary antibody, mouse anti-Oct4 (1∶100, Millipore) and rabbit anti-Ki67 (1∶500, Abcam) were diluted in PBS with 0.3% Triton-X100 and 0.5% BSA, and sections were incubated in a humid chamber at 4°C overnight, rinsed with PBS and incubated with goat anti-mouse Alexa Fluor^TM^ 488 or goat anti-rabbit secondary antibody Alexa Fluor^TM^ 555 (1∶500, Life Technologies) for 60 minutes at room temperature. After rinsing with PBS and co-staining with Hoechst 33342 (Life Technologies), coverslips were mounted using fluoromount (Sigma-Aldrich), and examined under fluorescent microscope.

### Statistics

When applicable one-way analysis of variance (ANOVA) followed by Tukey's multiple comparison post-hoc test was used to evaluate the statistical significance (*p<0.05) of the difference in values using the GraphPad Prism^TM^ software (GraphPad Software Inc).

## Results

### Maintenance of mouse embryonic stem cells

Feeder-dependent and feeder-independent cells were cultured on feeder cells or gelatin-coated surfaces respectively in SCM, mES Prime Kit medium, 2i medium or as free-floating spheroids in ESN2 and 2i media as described in *Material and Methods*. Feeder-free cells expanded in 2i medium did not maintain their adherent properties and after one or two passages the cells grew as free-floating spheroids. Uncoated plastic as well as standard coating procedures with gelatin, collagen-I, and fibronectin were tested, but none of the coatings could support adherence properly (fig. S1). However, 2% ES-qualified FBS added to the 2i medium allowed for complete cell attachment with subsequent mES characteristic colony formation and propagation, an effect that could not be repeated by using knock-out serum replacement KOSR (fig. S1). Lower levels of FBS, down to 1%, mediated almost a complete adherence; while cell attachment at lower levels was markedly decreased (data not shown). Hence, we decided to culture the mES cells in suspension in serum-free 2i medium, as well as adherently in 2i supplemented with 2% serum. In addition, to investigate whether the 2i media can be used to adapt feeder-dependent mES cell lines to grow in the absence of MEFs on gelatin, we cultured C57 cells adherently in 2i supplemented with 2% serum.

**Figure 1:**
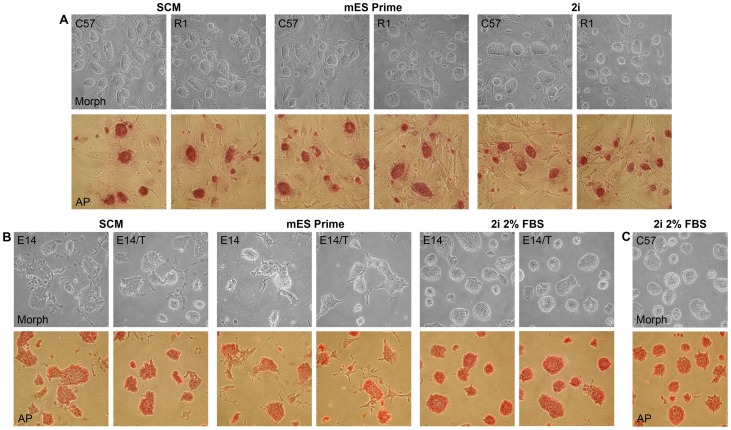
Adherent mouse ES cell colony morphology and alkaline phosphatase activity. Phase-contrast micrographs of colony morphology and alkaline phosphatase activity staining for mES cells grown on feeders (A) and mES cells grown on gelatin (B) for 15 passages in SCM, mES Prime Kit or 2i media. Noteworthy is that the feeder-cell dependent mES cell line C57 was maintained in 2i medium on gelatin for at least 15 passages without showing morphological signs of spontaneous differentiation or loss in alkaline phosphatase activity (C).

Morphologically the different media showed no apparent difference in maintaining feeder-dependent R1 and C57 cells on MEFs for 15 passages, at which stage the majority of colonies stained positive for AP activity (Fig. 1A). Feeder-independent E14 and E14/T cells grown on gelatin in SCM and mES Prime Kit exhibited similar colony morphology and AP staining along with a fraction of cells appearing partly differentiated (Fig. 1B). In contrast, feeder-free cultures grown in 2i supplemented with 2% serum exhibited exceedingly homogenous colonies with no signs of differentiation (Fig. 1B). Interestingly, the feeder-dependent C57 mES cell line was also successfully maintained for 15 passages on gelatin in 2i medium +2% FBS. Similar to E14 and E14/T cells, the C57 cells grew in dense compact colonies, showed no signs of spontaneous differentiation, and stained positive for AP activity (Fig. 1C).

**Figure 2:**
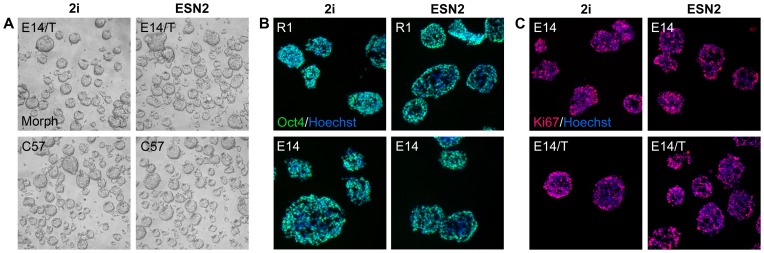
Phenotypc analysis of free-floating mouse ES cell sphere morphology as assessed by phase-contrast microscopy and immunochemistry. Phase-contrast micrographs of spheroid morphology (A) and fluorescence micrographs of Oct4 (B) and Ki67 (C) immunohistochemical detection of Oct4 and Ki67 in cryosectioned spheroids of mES cells grown in 2i or ESN2 media.

Cells grown in suspension formed homogenous looking spheroids (Fig. 2A). Spheroid sections stained positive Oct4 and Ki67, suggesting that the cells throughout these spheres are mitotically active self-renewing mES cells (Fig. 2B and 2C). All cell lines grown under the different culture conditions could be readily frozen and thawed again with no signs of excessive cell death or differentiation. After 15 passages an excess of 95% of the cells displayed an euploid karyotype (data not shown).

### Proliferation analysis

**Figure 3:**
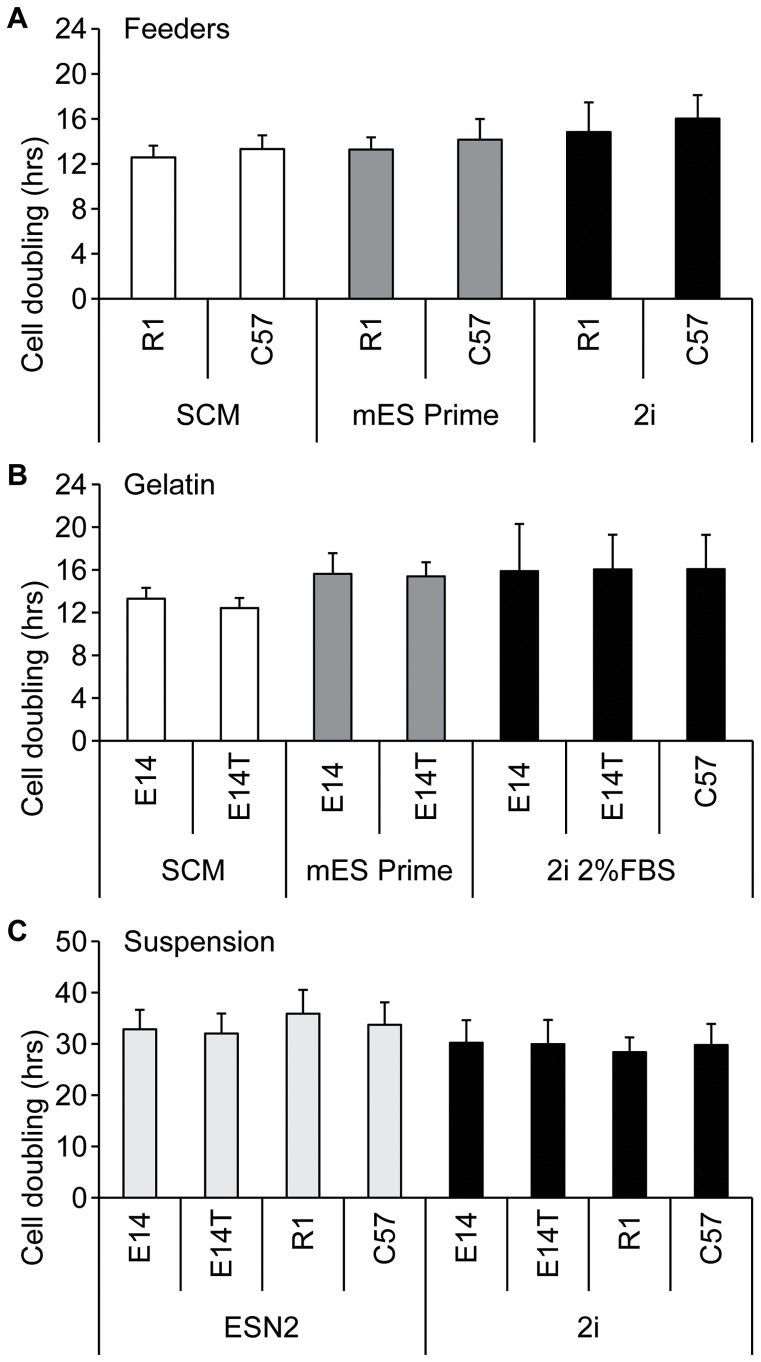
Proliferation rates of mouse ES cells cultured using different protocols. Cell doubling time assessment of mES cells grown on feeders (A) in SCM, mES Prime Kit and 2i media; on gelatin (B) in SCM, mES Prime Kit and 2i media supplemented with 2% FBS; or in suspension (C) in 2i and ESN2 media. Results are means ± sd for 5 consecutive passages (n = 3).

To assess the proliferation rate for each cell culture protocols the cell doubling times for 5 consecutive passages were assessed. R1 and C57 cells expanded on feeder cells exhibited a doubling time of around 13 h in SCM and mES Prime Kit medium and 15 h in 2i medium +2% FBS (Fig. 3A). E14 and E14/T cells cultured on gelatin showed doubling times of 13 h in SCM and 15–16 h in mES Prime Kit and 2i medium +2% FBS respectively but none of these differences were statistically significant. (Fig. 3B). However, all mES cell lines cultured in suspension in either 2i or ESN2 medium exhibited markedly increased doubling times of around 30 and 34 h, respectively (Fig. 3C). No significant difference was found when comparing the two suspension cultures.

### Gene Expression analysis

**Figure 4:**
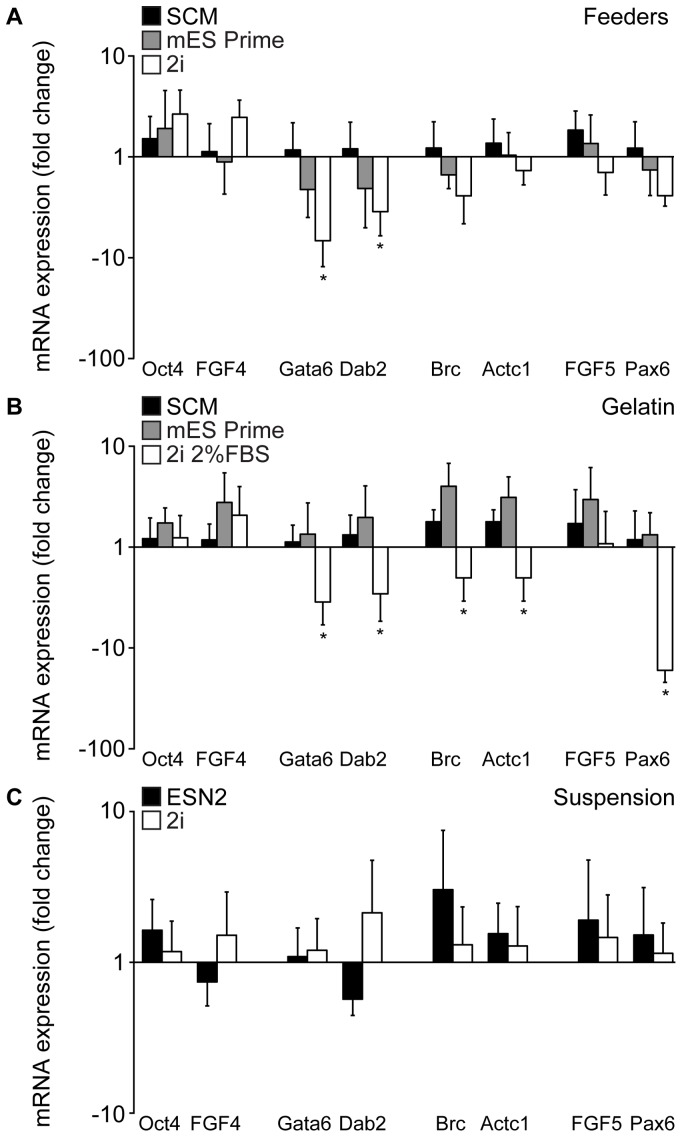
Analysis of ES cells cultured using different protocols as assessed by quantitative PCR. Quantitative PCR analysis at passage 15 for genes associated with undifferentiated mES cells, i.e. Oct4 and Fgf4, as well as early indicator genes for the three germ layers (endoderm: Dab2 and Gata6; ectoderm: Fgf5 and Pax6; mesoderm: Brc and Actc1). Graphs represent pooled results for mES cell lines grown on feeders (A) or on gelatin (B) in SCM, mES Prime Kit or 2i media, and in suspension (C) in 2i or ESN2 media. 18S expression is used for normalization, and results are comparative (SCM values for feeders and gelatin, and 2i media for suspension, were set as control) Ct value means ± sd (n = 3) p(*) <0.05.

After 15 passages under the previously defined conditions the expression of common genes specific for undifferentiated mES cells were examined by quantitative PCR. As expected, high levels of both Oct4 and FGF4 were observed in all culturing conditions and no clear difference was found between the four cell lines used (data not shown). When comparing the various growth media, cells grown on feeders in 2i medium exhibited an increase in both Oct4 and FGF4 expression compared to the cells grown in SCM and mES Prime Kit (Fig. 4A). This increase was however not statistically significant. Concomitantly with these data, an overall decrease; approximately 2-fold for the ectoderm (FGF5 and Pax6) and mesoderm markers (Brc and Actc1) and up to a significant 5-fold for the endoderm markers (Dab2 and Gata6), was detected (Fig. 4A). Also cell cultures grown in mES Prime Kit showed a slight decrease in spontaneous differentiation towards endoderm compared to SCM (Fig. 4A). In accordance with the cultures on MEFs, feeder-independent cells grown on gelatin in 2i medium +2% FBS showed a significant decrease in several of the investigated differentiation markers (Fig. 4B). There was no significant difference between the cells grown feeder-free in mES Prime Kit compared to SCM (Fig. 4B). No distinct difference could be detected when comparing cells grown in different media in suspension (Fig. 4C). Surprisingly, although the levels of Oct4 were similar when comparing the various culturing procedures with each other, we found that cells grown on feeders generally exhibited a low, but noticeably higher degree of spontaneous differentiation compared to cells grown on gelatin or in suspension (fig. S2). When comparing the gene expression levels in C57 cells cultured on MEFs or gelatin in 2i medium +2% FBS, no significant differences were detected (data not shown), suggesting that the C57 cells, which usually require feeders, have been adapted to a feeder-free environment and can be maintained on a gelatinous surface in 2i medium for a prolonged period of time.

**Figure 5:**
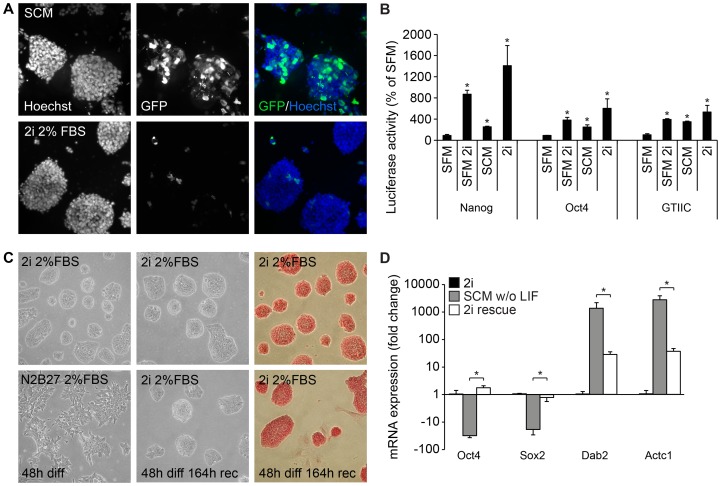
Characterisation of mouse ES cells cultured in 2i media. Fluorescence micrographs of GFP-expression E14 mES cells, pre-transfection cultured in 2i or SCM, transfected with Lipofectamine 2000 and pmaxGFP (A). Luciferase reporter assay for Oct4 and Nanog promoter activation, as well as Tead-enhanced promoter activation, 24 hrs post-transfection in E14 cells cultured without LIF in serum-free SCM with or without PD0325901 (1 μM) and CH99021 (3 μM), SCM, or 2i media. Results are mean ± sd (n = 3) p(*) <0.05 (B). Phase-contrast micrographs of colony morphology and alkaline phosphatase activity staining (C), and qPCR analysis (D) of E14 cell 96 hrs after 2i rescue and clean-up of partially differentiated mES cells cultures. 18S expression is used for normalization, and results are comparative (expression levels at passage 15 set as control) Ct value means ± sd (n = 3) p(*) <0.05.

To assess whether 2i medium specifically increases transcriptional activation of some of the core pluripotency regulators, e.g. Oct4, Nanog, and the recently reported TEAD/TEF family of transcription factors [24], [31], the cells were transfected with different luciferase reporter constructs as previously described [24]. We found, however, that cells that had been cultured in 2i medium were much harder to transfect using Lipofectamine 2000, a widely used transfection reagent to introduce plasmid DNA into mES cells, as compared to cells that had been cultured in SCM (Fig. 5A). Therefore, cells were cultured in SCM prior to transfection and at four hours post-transfection the media was changed to fresh serum-free medium with or without the 2i inhibitors, SCM or complete 2i medium and cultured for 24 hrs. SCM induced the lowest, albeit significant, increase in promoter activities compared to serum-free medium. Addition of two inhibitors to SFM further increased these activities, but the strongest promoter activity was obtained with complete 2i medium (Fig. 5B). Notably, when normalizing our luciferase values to transfection efficacy control we found that SCM induced a massive induction of beta-galactosidase activity compared to the 2i medium, which *per se* was not significantly higher than the beta-galactosidase activity measured under serum-free conditions (fig. S3). These results suggest that while serum induces transcription of many genes via a plethora of pathways, the 2i inhibitors specifically activate a limited number of critical pathways important for ES cell maintenance.

There are commercially available media that are marketed with having the ability to rescue ES cell cultures that have started to drift, e.g. RESGRO™ from Millipore. In a previous study a predecessor of the 2i medium using an additional inhibitor of the FGF receptor was shown to have a similar ability [23]. To investigate whether this ability can also be attributed to 2i media, we examined the capacity of 2i medium to rescue and “clean up” partially differentiated mES cell cultures. Cells were let to spontaneously differentiate in SCM without LIF for 48 h, and subsequently maintained for 6 days in 2i medium. AP-staining and morphological assessment showed that within 3 passages the rescued cultures were almost identical to control cultures, whereas no signs of ES cell typical colonies or AP-positive cells could be found in cultures that had been allowed to continue differentiating (Fig. 5C). This was further confirmed by qPCR analysis demonstrating that while cells that continued to differentiate showed a distinct down regulation of Oct4 and Sox2, cells rescued with 2i medium showed similar Oct4 and Sox2 levels as cells that had continuously been cultured in 2i medium (Fig. 5D). Although the rescued cultures expressed higher levels of Dab2 and Actc1 than control cultures, the levels were approximately 50 fold lower than in cultures that had continued to differentiate (Fig. 5D). The elevated levels of Dab2 and Actc1 in the rescued cultures are likely due to a small amount of differentiated cells remaining in the culture and a couple of more passages would eliminate these cells completely from the cultures.

### Differentiation capacity

**Figure 6:**
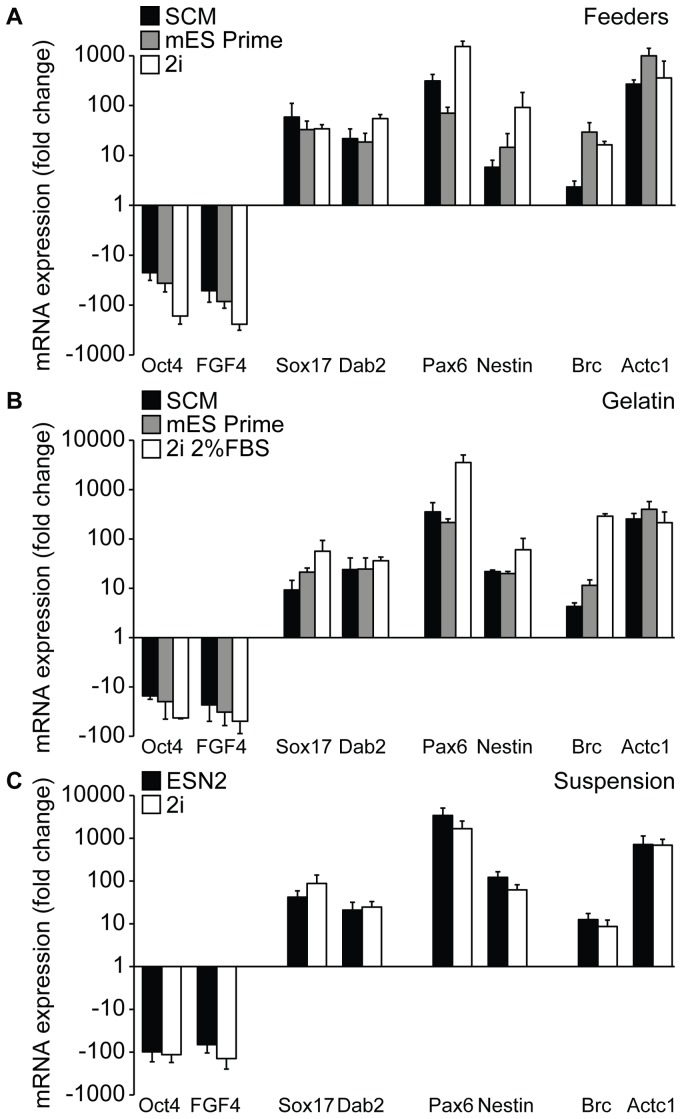
Analysis of ES cells induced to differentiate as assessed by quantitative PCR. Quantitative PCR analysis for the expression of Oct4 and FGF4 and indicator genes for the three germ layers in mES cells that were induced to differentiate for 6(A) or on gelatin (B) in SCM, mES Prime Kit or 2i media, and in suspension (C) in 2i or ESN2 media. 18S expression is used for normalization, and results are comparative (expression levels at passage 15 set as control) Ct value means ± sd (n = 3).

To confirm that all culture conditions support mES pluripotency, the cells were cultured for 15 passages in different media and then induced to differentiate in SCM without LIF for six days. Post-differentiation expression levels were normalized to the expression levels after 15 passages in the different media, and showed significantly reduced levels of Oct4 and FGF4 (Fig. 6A–C) in response to differentiation regardless of the protocol that had been used to maintain the cells. Additionally, significantly increased levels of all differentiation markers for the three germ layers could also be detected, showing that all the culture protocols could maintain mES cell pluripotency for an extended period of time (Fig. 6A–C). However, feeder-dependent ES cells generally exhibited a somewhat lower expression level of the differentiation markers as compared to cells grown on gelatin or in suspension, suggesting that differentiation in these cultures takes slightly longer. Overall no significant difference between the various growth media could be detected.

### Cost and time comparison

**Figure 7:**
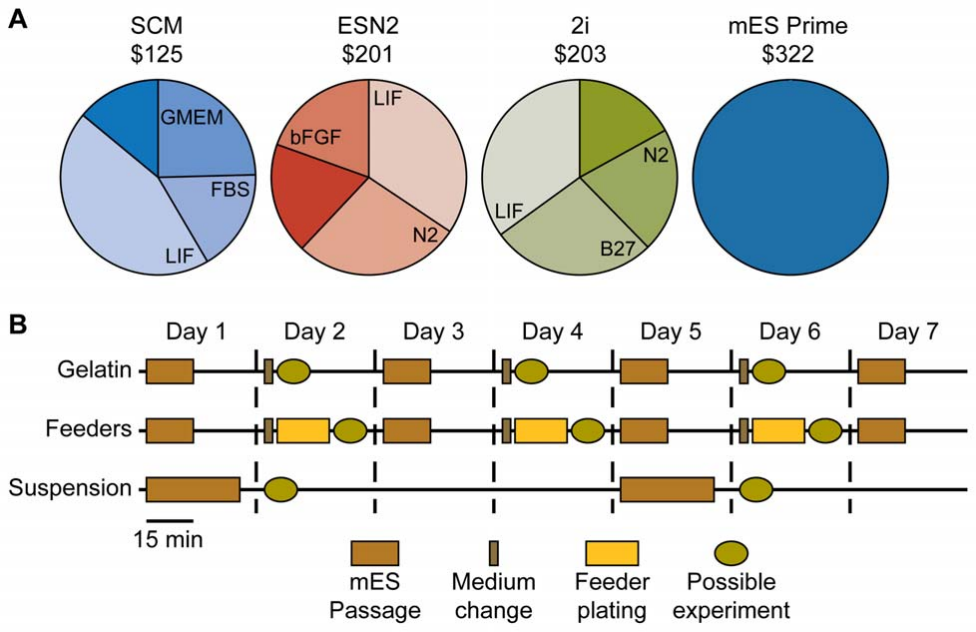
Cost and hands-on time for different ES cell culturing protocols. (A) Cost comparison and the three most expensive constituents for 500 ml ready-to-use SCM, 2i and ESN2 media (based on the list price for medium-sized bulk purchases), and the mES Prime Kit. (B) Estimated minimum cell maintenance hands-on time and possible experimental set-ups for seven days of mES cultures on feeders and gelatin, or in suspension.

In order to compare the costs for each medium we used the list price in the United States for medium-sized bulk purchases of each constituent of the three lab made media, i.e. SCM, 2i and ESN2, and calculated the price for 500 ml ready-to-use media. The cost for mES Prime Kit is the list price for one 500 ml bottle ready-to-use media. We deliberately discounted for the passaging cost since the difference between the various protocols are negligible. Also, we opted to remove the cost for feeder cells, since this cost is too difficult to assess as these can be bought ready-to-use, as proliferating cell lines that need to be mitotically inactivated, or most often made from scratch from mouse embryos. We have however added the cost for the feeder media, which in this study was used in a ratio of 1∶2 compared to mES cell media for feeder-dependent mES cell cultures. Our cost assessments (for details see table S1) show that the lab-made SCM (∼$125) is the most cost efficient, with the other lab-made media being about 60% more expensive at around $200 each (Fig. 7A). The largest expense is LIF, which makes up for more than 45% of the total SCM cost and for about 30% of ESN2 and 2i media costs. Although more than double the price of SCM, the commercially available medium mES Prime Kit (∼$322) consists of only two components that have been mES culture tested and thus serve as a convenient and time-saving alternative to lab-made media consisting of a multitude of components that need to be aliquoted, stored and tested for lot-to-lot variability. The cost for the N2 containing media (e.g. 2i and ESN2) can be reduced by approximately 5% and 10%, respectively if making the N2 supplement in-lab. In adherent cultures media are changed every day and cells passaged every other day, while the suspension cultures are passaged every four days and no extra media is added between passages. As shown above (figure 3) the cell doubling time for the suspension cultures are almost exactly twice as long as for the adherent cultures, and since there is no significant difference in cell doubling times between different culturing conditions for the adherent cultures, cell numbers are approximately similar for all cultures at cell passage. Hence, to better relate the cost we have compared the costs per passage for the various protocols and media (table S1). The passage costs are assessed for one 10-cm dish with 10 ml culture media, which is changed once for adherent cultures, and 5 ml for passaging procedures. Regardless of mES cell media, feeder-dependent cultures cost about $1 more per passage than feeder-independent cultures due to the extra 10 ml per passage MEF medium (∼$50 per 500 ml) needed for setting up the feeder layers (not including the cost of purchasing or making the feeders). The feeder-independent adherent cultures SCM cost approximately $6 per passage, which is considerably lower than the 2i media (∼$10) and the mES Prime Kit (∼$16). As media is not changed in the suspension cultures the cost for these cultures are 40% less than the adherent ones of approximately $6 for both the 2i and ESN2 media cultures. Although economically more sound, the net gains by using suspension cultures need to be weighed against the productivity (number of experiments that can be generated in a certain time) of the different protocols (see below).

As for ease of use, we estimated the minimum hands-on time during maintenance as well as the amount of possible experimental set-ups during 8 days of culture. Feeder-dependent mES cells takes approximately twice the amount of time to maintain (i.e. 2 hrs/week) as compared to feeder-free mES cells (1 hr/week) (Fig. 7B), which is mainly due to the extra time needed for thawing and setting up new feeder layers between each passage. Although maintaining suspension cultures takes less than one hour per week, the markedly reduced proliferation rate only allows for passaging of the cells every 4–5 days while the adherent cultures are split every second day. This means that the time spent per passage on suspension cultures are only slightly shorter than those spent on feeder-dependent cultures (i.e. both around 30 min/passage). The feeder-free cultures require half this time, and are thus, by far, the most time-efficient protocol. We should also point out that we found the suspension cultures are quite delicate to maintain. Although the cells before seeding were strained at each passage, we occasionally perceived the newly formed spheroid seeds to be adhesive and form large clusters. The cluster could be mechanically dispersed, but in our opinion not ideal for a mES cell standard maintenance procedure. Additionally, the more frequent passaging needed for adherent culture allows for more experimental set-ups. For the protocols described here, three times more cells can be produced every week using adherent cultures as compared suspension cultures.

## Discussion

In recent years several novel ES cell culture protocols have been published, and there are today a plethora of techniques and protocols in the literature claiming to be ideal and optimized for mES cell maintenance. Nevertheless, most groups still rely on old methods, which include growing the cells on mitotically inactivated MEFs in undefined media supplemented with FBS and LIF. In the present investigation we have compared the standard culture medium with two newly described ones; ESN2 and 2i. We found that mES cells detached after a passage when cultured feeder-free in 2i medium without serum. Gelatin, fibronectin and collagen coatings were tried, but none of these could promote cell attachment. Similar to cells grown in ESN2 with LIF and bFGF, the detached cells grew in spheroids that could be passaged and maintained for at least 15 passages without losing their pluripotency. Quantitative PCR analysis of the ESN2 and 2i suspension cultures revealed that the cells maintain high expression levels of pluripotency markers with very limited signs of spontaneously differentiation. These methods however have the disadvantage that the cells exhibit a slower growth rate and are quite tedious to maintain. The addition of 1–2% serum to the 2i cultures resulted in cells readily attaching and propagating in distinct colonies with little or no sign of spontaneous differentiation, which is commonly present (albeit at low frequency) in serum-dependent feeder-free mES cultures. Moreover, the proliferation rate increased and was similar to the other adherent culture protocols. To reduce serum levels in order to further lessen the culture costs is not recommended since levels below 1% did not promote complete mES cell attachment. We do not know which factor/s in serum that promote cell adhesion in 2i cultures but can exclude all components present in KOSR, since KOSR failed to promote attachment when added to the 2i medium. Moreover, serum does not seem to prime the cells to stay adherent since cells cultured in 2i medium with serum for several passages form suspension spheroids after their first passage upon serum removal (data not shown). Although the use of feeders cells for long has been one the key parameters that make *ex vivo* ES cell maintenance possible, their use is both time-consuming and adds to the costs. In addition, the unknown assortment of factors released by feeders may in worst-case cause difficulties in result interpretation. Therefore, to facilitate normal maintenance and experiments, a successful adaptation to feeder-free cultures is of great interest for many groups working in the stem cell field. The procedure can however be both tedious and gruelling, and several wholesalers offer expensive media solutions and kits that supposedly do this. Based on our results with feeder-dependent C57 cells grown in 2i media on gelatin we propose that 2i medium can readily be used to adapt feeder-dependent mES cell lines to be maintained on feeder-free surfaces. In our hands, the adaption seems to be instantaneous with little or no sign of cell death. A couple of passages are however needed to get rid of all feeder cells.

There were no significant differences in proliferation rates across the various mES cell lines and media in adherent cultures. Noteworthy, we see that most germ layer lineage specific genes that we analyze for are expressed, albeit at low levels, in serum-containing cultures on both feeders and gelatin but that these are significantly repressed in 2i cultures. This observation is in consort with a recent study by Marks and colleagues, in which they show that ectodermal and mesodermal specification genes are significantly decreased and pluripotency genes are slightly up-regulated in 2i cultures compared to cells cultured in serum [32]. They go on and show that despite that 2i medium and serum cultures have comparable differentiation potential, the cells have distinct gene expression profiles. It is proposed that the lineage-affiliated genes that are expressed in mES cells cultured in serum are in fact induced by serum rather than being an intrinsic trait of ES cells as previously perceived. This is in line with our pluripotency luciferase reporter data showing that the 2i inhibitors activates a few core transcription factors in contrast to LIF and serum that together induce many parallel pathways and stimulate cell growth.

Overall our data and observations confirm that all media used in the present benchmarking study maintain mES cell self-renewal and pluripotency (for overview see table S2), but with the 2i medium being somewhat superior due to its repression of spontaneous differentiation. The adherent culturing procedures increases the proliferation rate and thereby increasing the number of potential experimental set-ups per week and growing the cells feeder-free is easier, more cost-effective and less time-consuming. We show that feeder-dependent mES cell lines rapidly can be adapted to grow adherent and feeder-free in 2i media and that this medium can be used to rescue cultured that have started to differentiate. The means of blunting ES cells to exogenous factor-induced signalling in order to maintain them in a naïve ES cell state presently seem be the optimal approach for mES cell culturing. Although, the 2i medium most likely can be modified and economized, e.g. the antioxidants and free-radical scavengers proposed to be the imperative elements in the B27 supplement [16] might be replaceable and substituted by a single factor. The most expensive constituent of N2 supplement is by far transferrin, which is a universal iron carrier that helps cells maintain homeostasis by regulating iron uptake. The other components such as insulin, which helps cells to use glucose and amino acids, and selenium, an integral part of several redox enzymes, are not as expensive but no less important for the substitution of serum. These factors are commonly sold as ITS supplements and the price, which can be as low as 10–20% compared to N2, varies based on the formulation. The missing components; BSA fraction V, progesterone and putrecine are all inexpensive and easy to add. However, a drawback with the 2i media is that it generates cultures that are very difficult to transfect, perhaps due to the dense nature of the formed colonies, why we cultured the cells for one passage in a standard serum-containing medium with LIF prior to transfection.

## Supporting Information

Figure S1 **Mouse ES cell adherence in 2i media.** Phase-contrast micrographs of colony/spheroid morphology of E14 mES cells seeded onto gelatin, fibronectin, or collagen (A) and cultured in 2i, and (B) on gelatin in 2i media supplemented with either 2% FBS or 2% KOSR.
(TIF)

Figure S2 **Analysis of ES cells as assessed by quantitative PCR.** Quantitative PCR analysis at passage 15 for genes associated with undifferentiated mES cells, i.e. Oct4 and Fgf4, as well as early indicator genes for the three germ layers (endoderm: Dab2 and Gata6; ectoderm: Fgf5 and Pax6; mesoderm: Brc and Actc1). Graphs represent pooled results for mES cell lines grown on feeders and gelatin in SCM, mES Prime Kit or 2i media, or in suspension in 2i or ESN2 media. 18S expression is used for normalization, and results are comparative (mES cultures on feeders in SCM set as control) Ct value means ± sd (n = 3).
(TIF)

Figure S3 **Beta-galactosidase activity in mouse ES cells cultured in SCM or 2i media.** Corresponding beta-galactosidase activity for luciferase activity measurements presented in Fig 5B 24 hrs post-transfection in E14 cells cultured without LIF in serum-free SCM with or without PD0325901 (1 μM) and CH99021 (3 μM), SCM, or 2i media. Results are mean ± sd (n = 3) p(*) <0.05.
(TIF)

Table S1 **Cost assessment of the SCM, ESN2, 2i, MEF media and lab-made N2 supplement.** The table shows the costs rounded to the nearest dollar based on the list price for the United States as stated by the respective supplier, and are calculated for 500 ml of ready made media or per passage in a 10-cm cell culture dish with 10 ml media.
(TIF)

Table S2 **Summary of the investigated parameters.** The table shows an overview of the obtained results for the various culturing regimes used in the present study. Since all regimes have been shown to be sufficient for adequate mES cell maintenance, they have been rated with one to three plus (+) signs with the three being topmost.
(TIF)

## References

[1] Martin GR (1981) Isolation of a pluripotent cell line from early mouse embryos cultured in medium conditioned by teratocarcinoma stem cells. Proc Natl Acad Sci USA 78: 7634–7638. 6950406 10.1073/pnas.78.12.7634 PMC349323

[2] Evans MJ, Kaufman MH (1981) Establishment in culture of pluripotential cells from mouse embryos. Nature 292: 154–156. 7242681 10.1038/292154a0

[3] Meng GL, Zur Nieden NI, Liu SY, Cormier JT, Kallos MS, et al (2008) Properties of murine embryonic stem cells maintained on human foreskin fibroblasts without LIF. Mol Reprod Dev 75: 614–622. 17886269 10.1002/mrd.20790

[4] Eiselleova L, Peterkova I, Neradil J, Slaninova I, Hampl A, et al (2008) Comparative study of mouse and human feeder cells for human embryonic stem cells. Int J Dev Biol 52: 353–363. 18415935 10.1387/ijdb.082590le

[5] Smith AG, Heath JK, Donaldson DD, Wong GG, Moreau J, et al (1988) Inhibition of pluripotential embryonic stem cell differentiation by purified polypeptides. Nature 336: 688–690. 3143917 10.1038/336688a0

[6] Williams RL, Hilton DJ, Pease S, Willson TA, Stewart CL, et al (1988) Myeloid leukaemia inhibitory factor maintains the developmental potential of embryonic stem cells. Nature 336: 684–687. 3143916 10.1038/336684a0

[7] Smith AG (1991) Culture and differentiation of embryonic stem cells. Journal of Tissue Culture Methods 13: 89–94.

[8] Magin TM, McWhir J, Melton DW (1992) A new mouse embryonic stem cell line with good germ line contribution and gene targeting frequency. Nucleic Acids Res 20: 3795–3796. 1641353 10.1093/nar/20.14.3795 PMC334045

[9] Cheng J, Dutra A, Takesono A, Garrett-Beal L, Schwartzberg PL (2004) Improved generation of C57BL/6J mouse embryonic stem cells in a defined serum-free media. Genesis 39: 100–104. 15170695 10.1002/gene.20031

[10] Ying QL, Nichols J, Chambers I, Smith A (2003) BMP induction of Id proteins suppresses differentiation and sustains embryonic stem cell self-renewal in collaboration with STAT3. Cell 115: 281–292. 14636556 10.1016/s0092-8674(03)00847-x

[11] Price PJ, Goldsborough MD, Tilkins ML (1998) Embryonic stem cell serum replacement. International Patent Application WO 98/30679.

[12] Chaudhry MA, Vitalis TZ, Bowen BD, Piret JM (2008) Basal medium composition and serum or serum replacement concentration influences on the maintenance of murine embryonic stem cells. Cytotechnology 58: 173–179. 19101815 10.1007/s10616-008-9177-5 PMC2652552

[13] Andang M, Moliner A, Doege CA, Ibanez CF, Ernfors P (2008) Optimized mouse ES cell culture system by suspension growth in a fully defined medium. Nat Protoc 3: 1013–1017. 18536648 10.1038/nprot.2008.65

[14] Moliner A, Enfors P, Ibanez CF, Andang M (2008) Mouse embryonic stem cell-derived spheres with distinct neurogenic potentials. Stem Cells Dev 17: 233–243. 18447639 10.1089/scd.2007.0211

[15] Tropepe V, Hitoshi S, Sirard C, Mak TW, Rossant J, et al (2001) Direct neural fate specification from embryonic stem cells: a primitive mammalian neural stem cell stage acquired through a default mechanism. Neuron 30: 65–78. 11343645 10.1016/s0896-6273(01)00263-x

[16] Ying QL, Wray J, Nichols J, Batlle-Morera L, Doble B, et al (2008) The ground state of embryonic stem cell self-renewal. Nature 453: 519–523. 18497825 10.1038/nature06968 PMC5328678

[17] Kunath T, Saba-El-Leil MK, Almousailleakh M, Wray J, Meloche S, et al (2007) FGF stimulation of the Erk1/2 signalling cascade triggers transition of pluripotent embryonic stem cells from self-renewal to lineage commitment. Development 134: 2895–2902. 17660198 10.1242/dev.02880

[18] Stavridis MP, Lunn JS, Collins BJ, Storey KG (2007) A discrete period of FGF-induced Erk1/2 signalling is required for vertebrate neural specification. Development 134: 2889–2894. 17660197 10.1242/dev.02858

[19] Wray J, Kalkan T, Gomez-Lopez S, Eckardt D, Cook A, et al (2011) Inhibition of glycogen synthase kinase-3 alleviates Tcf3 repression of the pluripotency network and increases embryonic stem cell resistance to differentiation. Nat Cell Biol 13: 838–845. 21685889 10.1038/ncb2267 PMC3160487

[20] Buehr M, Meek S, Blair K, Yang J, Ure J, et al (2008) Capture of authentic embryonic stem cells from rat blastocysts. Cell 135: 1287–1298. 19109897 10.1016/j.cell.2008.12.007

[21] Hanna J, Markoulaki S, Mitalipova M, Cheng AW, Cassady JP, et al (2009) Metastable pluripotent states in NOD-mouse-derived ESCs. Cell Stem Cell 4: 513–524. 19427283 10.1016/j.stem.2009.04.015 PMC2714944

[22] Nichols J, Jones K, Phillips JM, Newland SA, Roode M, et al (2009) Validated germline-competent embryonic stem cell lines from nonobese diabetic mice. Nat Med 15: 814–818. 19491843 10.1038/nm.1996

[23] Kiyonari H, Kaneko M, Abe S, Aizawa S (2010) Three inhibitors of FGF receptor, ERK, and GSK3 establishes germline-competent embryonic stem cells of C57BL/6N mouse strain with high efficiency and stability. Genesis 48: 317–327. 20162675 10.1002/dvg.20614

[24] Tamm C, Bower N, Anneren C (2011) Regulation of mouse embryonic stem cell self-renewal by a Yes-YAP-TEAD2 signaling pathway downstream of LIF. J Cell Sci 124: 1136–1144. 21385842 10.1242/jcs.075796

[25] Tong C, Huang G, Ashton C, Li P, Ying QL (2011) Generating gene knockout rats by homologous recombination in embryonic stem cells. Nat Protoc 6: 827–844. 21637202 10.1038/nprot.2011.338 PMC3855261

[26] Nagy A, Rossant J, Nagy R, Abramow-Newerly W, Roder JC (1993) Derivation of completely cell culture-derived mice from early-passage embryonic stem cells. Proc Natl Acad Sci U S A 90: 8424–8428. 8378314 10.1073/pnas.90.18.8424 PMC47369

[27] Holmborn K, Ledin J, Smeds E, Eriksson I, Kusche-Gullberg M, et al (2004) Heparan sulfate synthesized by mouse embryonic stem cells deficient in NDST1 and NDST2 is 6-O-sulfated but contains no N-sulfate groups. J Biol Chem 279: 42355–42358. 15319440 10.1074/jbc.C400373200

[28] Okumura-Nakanishi S, Saito M, Niwa H, Ishikawa F (2005) Oct-3/4 and Sox2 regulate Oct-3/4 gene in embryonic stem cells. J Biol Chem 280: 5307–5317. 15557334 10.1074/jbc.M410015200

[29] Hattori N, Imao Y, Nishino K, Ohgane J, Yagi S, et al (2007) Epigenetic regulation of Nanog gene in embryonic stem and trophoblast stem cells. Genes Cells 12: 387–396. 17352742 10.1111/j.1365-2443.2007.01058.x

[30] Jiang SW, Eberhardt NL (1995) Involvement of a protein distinct from transcription enhancer factor-1 (TEF-1) in mediating human chorionic somatomammotropin gene enhancer function through the GT-IIC enhanson in choriocarcinoma and COS cells. J Biol Chem 270: 13906–13915. 7775450 10.1074/jbc.270.23.13906

[31] Lian I, Kim J, Okazawa H, Zhao J, Zhao B, et al (2010) The role of YAP transcription coactivator in regulating stem cell self-renewal and differentiation. Genes Dev 24: 1106–1118. 20516196 10.1101/gad.1903310 PMC2878649

[32] Marks H, Kalkan T, Menafra R, Denissov S, Jones K, et al (2012) The transcriptional and epigenomic foundations of ground state pluripotency. Cell 149: 590–604. 22541430 10.1016/j.cell.2012.03.026 PMC3398752

