# Supplementary figures and images for: A Comparative Study of Protocols for Mouse Embryonic Stem Cell Culturing
Source: PLoS One. 2013 Dec 10;8(12):e81156. doi: 10.1371/journal.pone.0081156 (PMC3858223; doi:10.1371/journal.pone.0081156)

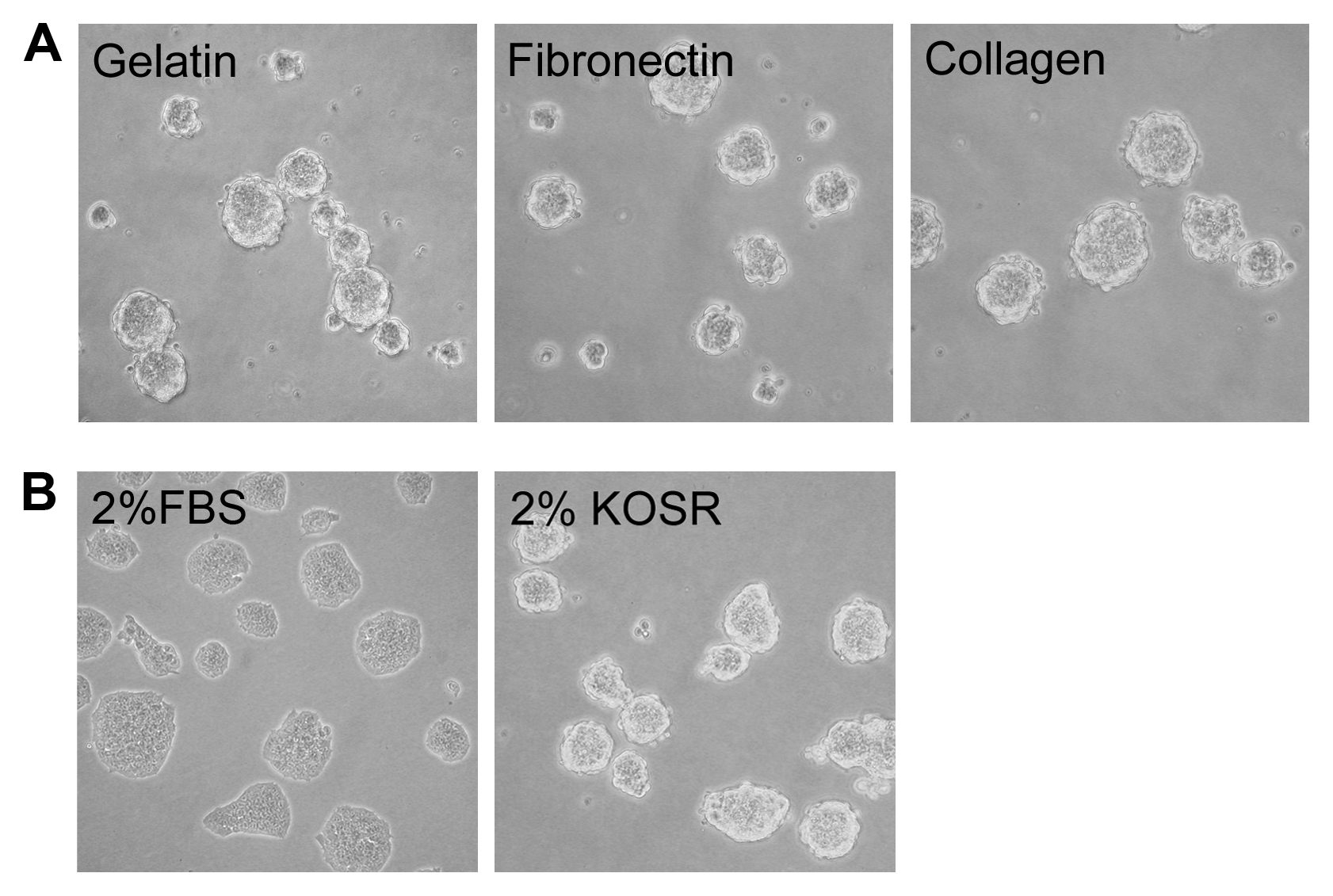

Supplement: Figure S1 — Mouse ES cell adherence in 2i media. Phase-contrast micrographs of colony/spheroid morphology of E14 mES cells seeded onto gelatin, fibronectin, or collagen (A) and cultured in 2i, and (B) on gelatin in 2i media supplemented with either 2% FBS or 2% KOSR. (TIF) [file pone.0081156.s001.tif]

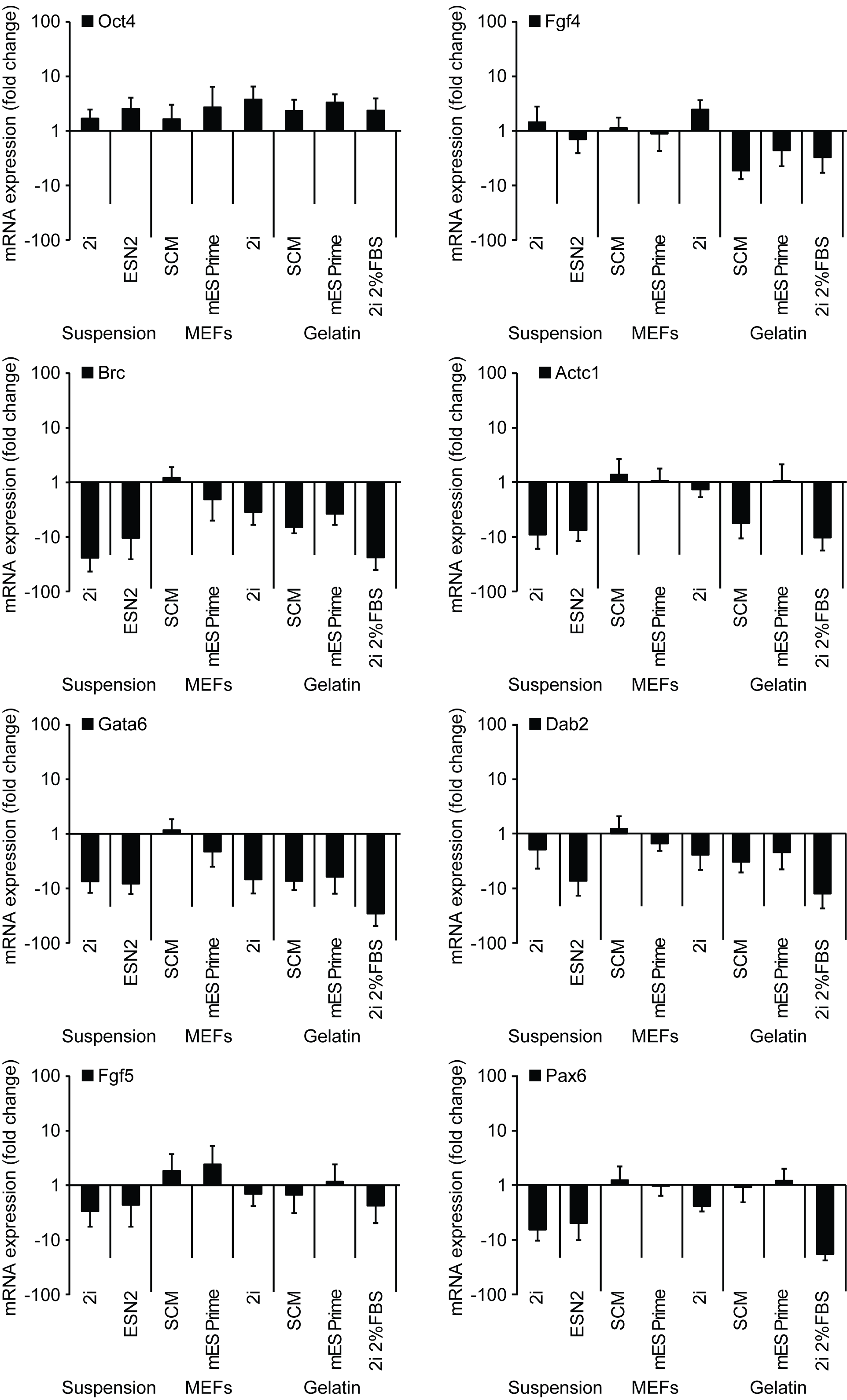

Supplement: Figure S2 — Analysis of ES cells as assessed by quantitative PCR. Quantitative PCR analysis at passage 15 for genes associated with undifferentiated mES cells, i.e. Oct4 and Fgf4, as well as early indicator genes for the three germ layers (endoderm: Dab2 and Gata6; ectoderm: Fgf5 and Pax6; mesoderm: Brc and Actc1). Graphs represent pooled results for mES cell lines grown on feeders and gelatin in SCM, mES Prime Kit or 2i media, or in suspension in 2i or ESN2 media. 18S expression is used for normalization, and results are comparative (mES cultures on feeders in SCM set as control) Ct value means ± sd (n = 3). (TIF) [file pone.0081156.s002.tif]

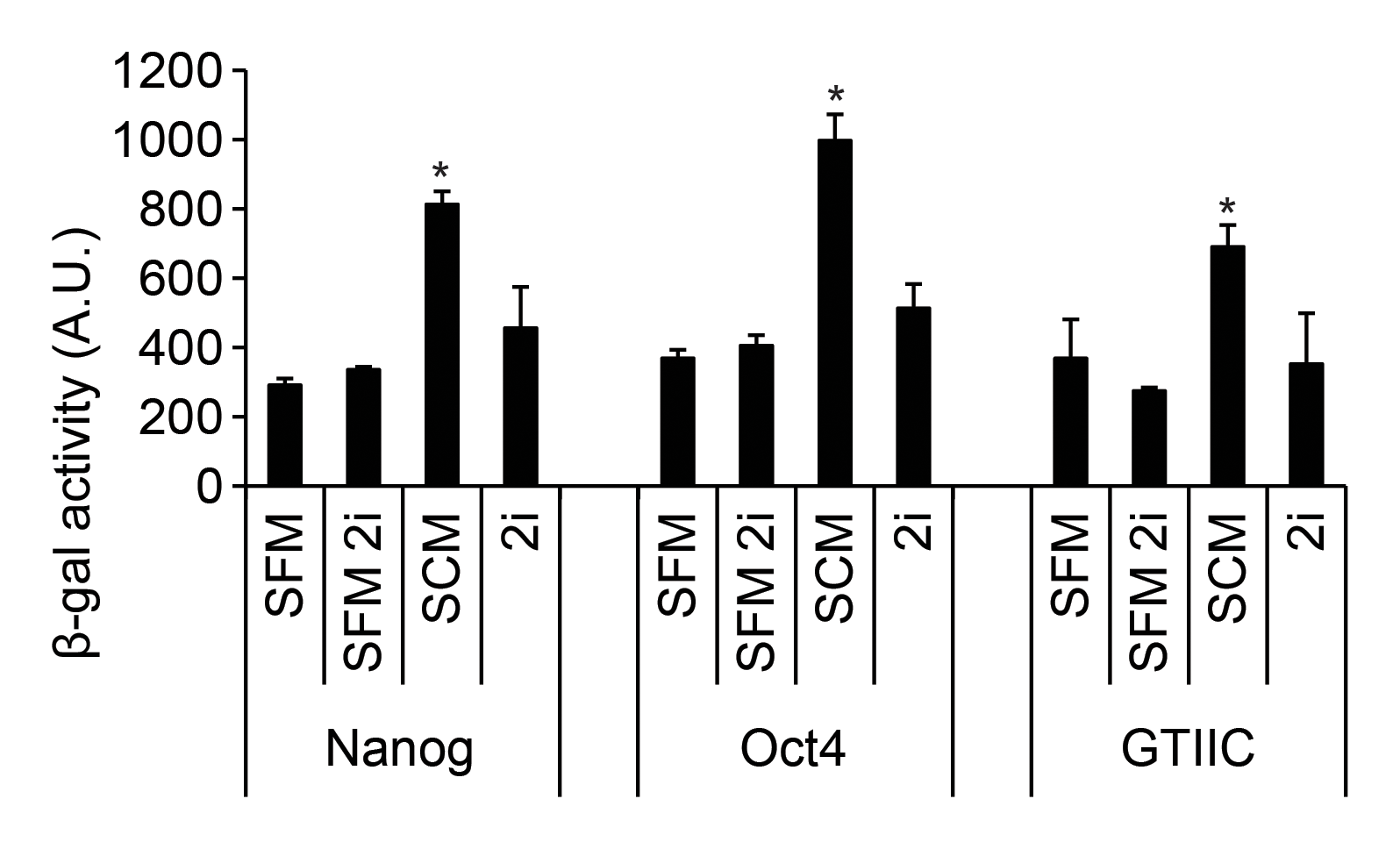

Supplement: Figure S3 — Beta-galactosidase activity in mouse ES cells cultured in SCM or 2i media. Corresponding beta-galactosidase activity for luciferase activity measurements presented in Fig 5B 24 hrs post-transfection in E14 cells cultured without LIF in serum-free SCM with or without PD0325901 (1 μM) and CH99021 (3 μM), SCM, or 2i media. Results are mean ± sd (n = 3) p(*) <0.05. (TIF) [file pone.0081156.s003.tif]

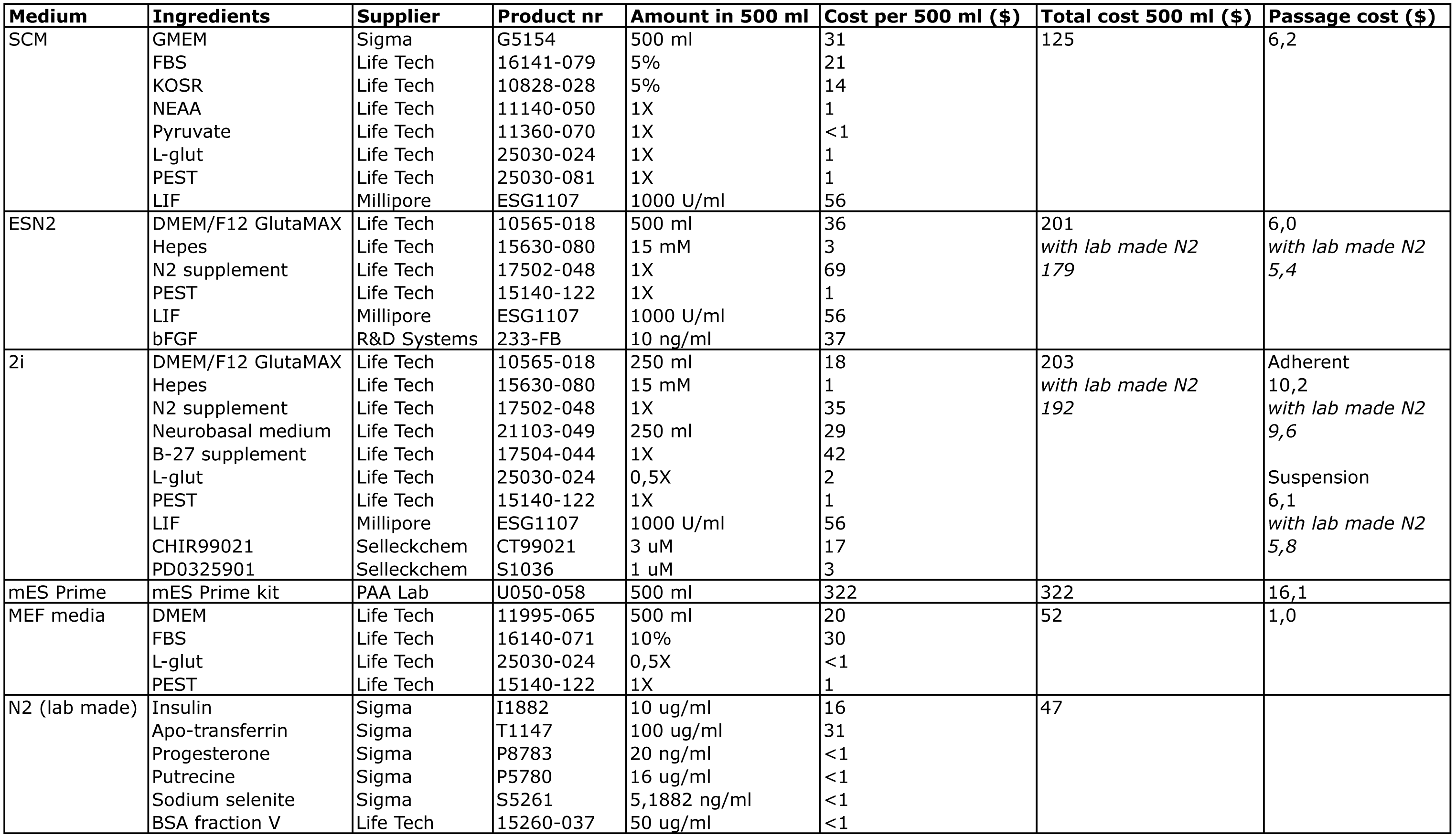

Supplement: Table S1 — Cost assessment of the SCM, ESN2, 2i, MEF media and lab-made N2 supplement. The table shows the costs rounded to the nearest dollar based on the list price for the United States as stated by the respective supplier, and are calculated for 500 ml of ready made media or per passage in a 10-cm cell culture dish with 10 ml media. (TIF) [file pone.0081156.s004.tif]

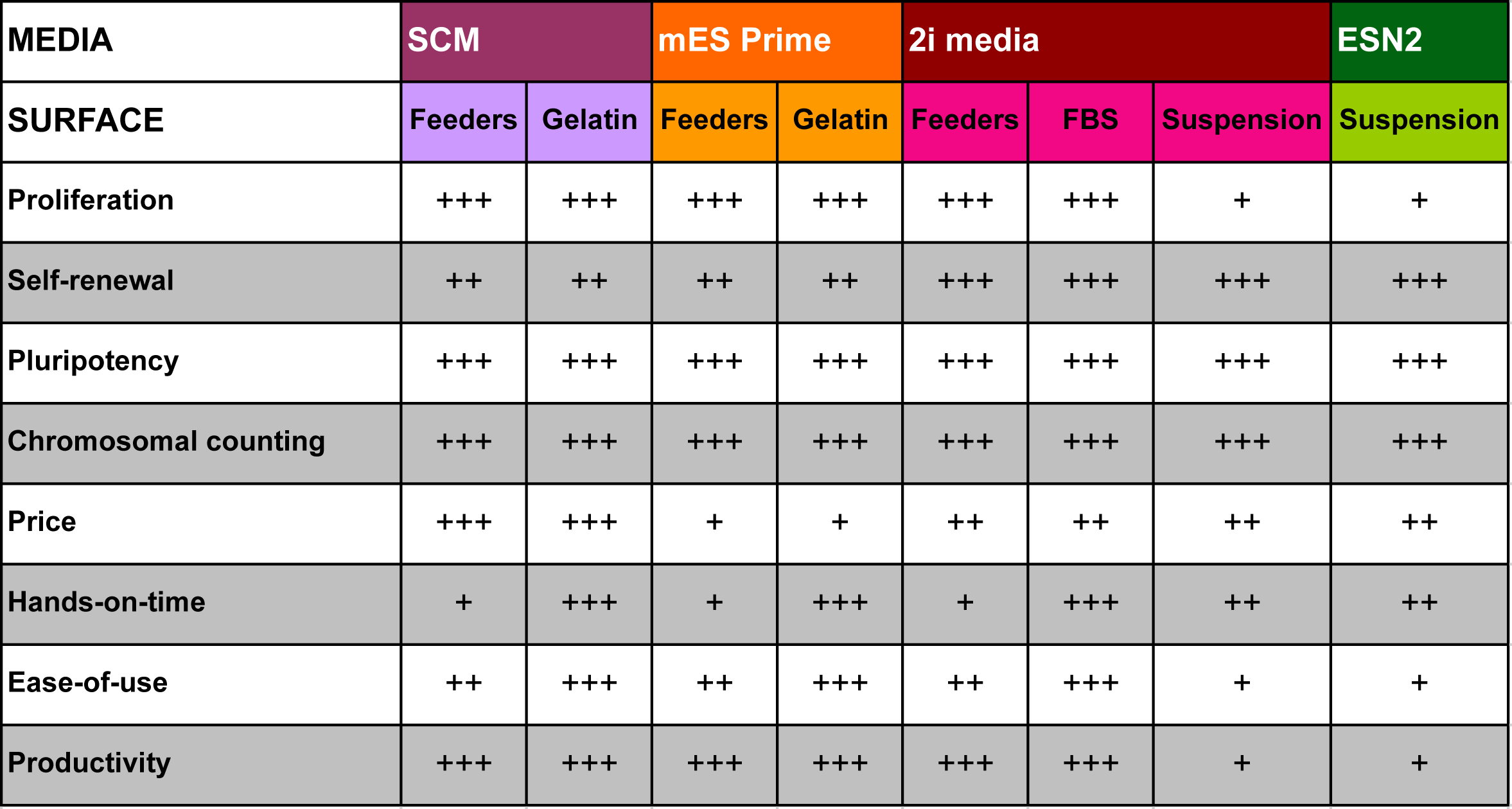

Supplement: Table S2 — Summary of the investigated parameters. The table shows an overview of the obtained results for the various culturing regimes used in the present study. Since all regimes have been shown to be sufficient for adequate mES cell maintenance, they have been rated with one to three plus (+) signs with the three being topmost. (TIF) [file pone.0081156.s005.tif]
